# Post-Hospital Care Intensity Modifies the Relationship Between Depressive Symptoms and Hospital Readmissions

**DOI:** 10.1093/geroni/igaa057.3317

**Published:** 2020-12-16

**Authors:** Leah Abrams, Geoff Hoffman

**Affiliations:** 1 University of Michigan School of Public Health, Ann Arbor, Michigan, United States; 2 University of Michigan School of Nursing, Ann Arbor, Michigan, United States

## Abstract

The objective of this study was to examine whether more structured post-acute care reduces the deleterious impact of depressive symptoms on older adults’ post-hospital outcomes (30-day hospital readmissions, 30-day falls, 1-year falls, and 1-year mortality). The sample comprised 23,485 eligible index hospitalizations from 7,151 unique fee-for-service Medicare beneficiaries from Health and Retirement Study linked to Medicare claims from 2000 to 2014. Depressive symptoms were measured using the eight-item Center for Epidemiologic Scale - Depression. We ran multinomial probit models regressing post-hospital setting on depressive symptoms while adjusting for sociodemographic factors, socioeconomic factors, family support, and health status. Then, we ran adjusted logistic regression models of each outcome while interacting depressive symptoms with post-acute care setting. We found that 62% of hospitalizations were routine discharges home, 17% were discharged to home health, and 21% were discharged to a Skilled Nursing Facilities (SNF). When adjusting for sociodemographic and socioeconomic factors, each increasing depressive symptom was associated with a half percentage point higher probability of referral to home health and 1.6 percentage points higher probability of discharge to SNFs, driven by differences in family support and health status. Rehabilitation in SNFs, compared to routine discharges home, reduced the positive association between depressive symptoms and 30-day readmissions (OR=0.95, p=0.027). However, post-acute care settings did not modify the association of depressive symptoms with falls or mortality. Considering depressive symptoms in discharge decisions, above and beyond their associations with family support or health status, may help hospitals avoid readmissions but may not improve health and functioning.

